# Predisposition to cortical neurodegenerative changes in brains of hypertension prone rats

**DOI:** 10.1186/s12967-023-03916-y

**Published:** 2023-01-27

**Authors:** Moti Ben-Shabat, Yaseen Awad-Igbaria, Shifra Sela, Bella Gross, Yoram Yagil, Chana Yagil, Eilam Palzur

**Affiliations:** 1 Research Institute of Galilee Medical Center, Nahariya, Israel; 2 Azrieli Faculty of Medicine, Bar-Ilan University, Safed, Israel; 3 Neurology Department, Galilee Medical Center, Nahariya, Israel; 4 Laboratory for Molecular Medicine, Barzilai University Medical Center, Ashkelon, Israel; 5 Faculty of Health Sciences, Ben-Gurion University of the Negev, Beer-Sheba, Israel

**Keywords:** Genetic predisposition, Hypertension, Dietary Salt Loading, Neurodegeneration, SBH/y, SBN/y

## Abstract

**Background:**

Substantial evidence suggests that hypertension is a significant risk factor for cognitive decline. However, it is unclear whether the genetic predisposition to hypertension is also associated with cellular dysfunction that promotes neurodegeneration.

**Methods:**

Changes in blood pressure were evaluated following dietary salt-loading or administration of a regular diet in Sabra Normotensive (SBN/y) and Sabra Hypertension-prone rats (SBH/y). We performed quantitative RT-PCR and immunofluorescence staining in brain cortical tissues before salt loading and 6 and 9 months after salt loading. To examine the expression of brain cortical proteins involved in the gene regulation (Histone Deacetylase-HDAC2; Histone Acetyltransferase 1-HAT1), stress response (Activating Transcription Factor 4-ATF4; Eukaryotic Initiation Factor 2- eIF2α), autophagy (Autophagy related 4A cysteine peptidase- Atg4a; light-chain 3-LC3A/B; mammalian target of rapamycin complex 1- mTORC1) and apoptosis (caspase-3).

**Results:**

Prior to salt loading, SBH/y compared to SBN/y expressed a significantly higher level of cortical HAT1 (protein), Caspase-3 (mRNA/protein), LC3A, and ATF4 (mRNA), lower levels of ATG4A (mRNA/protein), LC3A/B, HDAC2 (protein), as well as a lower density of cortical neurons. Following dietary salt loading, SBH/y but not SBN/y developed high blood pressure. In hypertensive SBH/y, there was significant upregulation of cortical HAT1 (protein), Caspase-3 (protein), and eIF2α ~ P (protein) and downregulation of HDAC2 (protein) and mTORC1 (mRNA), and cortical neuronal loss.

**Conclusions:**

The present findings suggest that genetic predisposition to hypertension is associated in the brain cortex with disruption in autophagy, gene regulation, an abnormal response to cellular stress, and a high level of cortical apoptosis, and could therefore exacerbate cellular dysfunction and thereby promote neurodegeneration.

**Supplementary Information:**

The online version contains supplementary material available at 10.1186/s12967-023-03916-y.

## Background

Despite considerable success in treatment and prevention, hypertension is still one of the leading causes of morbidity and mortality worldwide [1, 2]. Globally, it has been estimated that hypertension affects 30–45% of the adult population [3]. Hypertension is a complex multifactorial disease that results from an interaction between genetic predispositions [4, 5], environmental risk factors (e.g., smoking, poor diet, high sodium consumption, overweight and obesity, alcohol consumption, physical inactivity, and sedentary lifestyles), and psychological and social factors (e.g., stress, anxiety, depression, and low socioeconomic status) [6–9].

Hypertension in its present form continues to cause target organ damage, including the brain, heart, kidneys, and vasculature. In the brain, hypertension contributes to the development of neurodegenerative diseases that manifest in dementia and cognitive impairment [10, 11]. Neurodegenerative diseases are associated with well-documented morphological and anatomical brain changes in humans and in animal models, including white matter lesions, reduced neuronal connectivity, nerve cell number, and decreased cortical volume [12–19]. Moreover, these brain changes are directly associated with cognitive impairment and dementia [12–14, 16, 17, 20–22].

Hypertension has been associated with impaired cellular functions such as autophagy, apoptosis, transcription regulation, and stress response, all of which have been associated with neurodegeneration and dementia [23–25]. We have recently shown that hypertension in a rat model affects the autophagy process, the primary pathway for the degradation of entire organelles and macromolecules in the lysosome, and acts as an adaptive mechanism essential for maintaining cell survival under different stress conditions [23]. We have also shown that hypertension decreases autophagy-related 4A cysteine peptidase (Atg4a) in the rat cortex [23]. The Atg4 family plays a critical function in autophagosome formation through many proteins, including the essential LC3A and LC3A/B, which are involved in the normal homeostasis of misfolded proteins degradation [20]. Thus, it could be assumed that interruption in the autophagy process might contribute to the activation of the apoptosis pathway and neuronal death [26–28]. Indeed, elevated levels of neuronal apoptosis have been observed in an animal model of hypertension [23].

Moreover, previous studies have shown that hypertension induces considerable alterations in the regulation and expression of genes in the brain cortex. For instance, as a result of environmental and physiological stress, such as imposed by hypertension, leads to an imbalance in gene regulation by acetylation and deacetylase [23]; in addition, under cellular stress, the Eukaryotic Initiation Factor 2 (eIF2α) phosphorylation represses global translation coincident with a preferential translation of Activating Transcription Factor 4 (ATF4), a master regulator controlling the transcription of key genes essential for adaptative functions [29, 30].

The interaction between genetic predisposition to hypertension and environmental factors contributes to the development of hypertension. The increase in blood pressure promotes alteration in gene expression, dysfunction in the autophagy process, and epigenetic modification in neuronal cells, leading to neuronal stress response or cell death and apoptosis, resulting in brain morphological and anatomical changes associated with neurodegeneration and dementia. It is still unclear, however, whether genetic predisposition to hypertension may already be associated with cell dysfunction involved in neurodegeneration development. In the current study, we investigated in an inbred rodent model genetically predisposed to salt-sensitive hypertension the long-term effect of an environmental factor (high salt dietary intervention) on an array of neuronal cell functions that have been related to neurodegeneration and dementia.

## Methods

### Animals

Three-month-old male Sabra Hypertension-prone (SBH/y) and Sabra Hypertensive-resistant (SBN/y) rats were used in this study as a model of salt-sensitive or resistant hypertension, respectively [31, 32]. The animals were procured from the Israeli Rat Genome Center (Ashkelon, Israel). All animal procedures were approved by the Bar-Ilan University Animal Care Committee (Code# 57-12-2014) and were carried out in accordance with the National Institutes of Health Guide for the Care and Use of Laboratory Animals. During the study, animals were housed in groups of 3–4 rats in a sterilized solid bottom cage with contact bedding under controlled temperature and a 12:12 h light/dark cycle.

### Animal model of hypertension

**Fig. 1:**
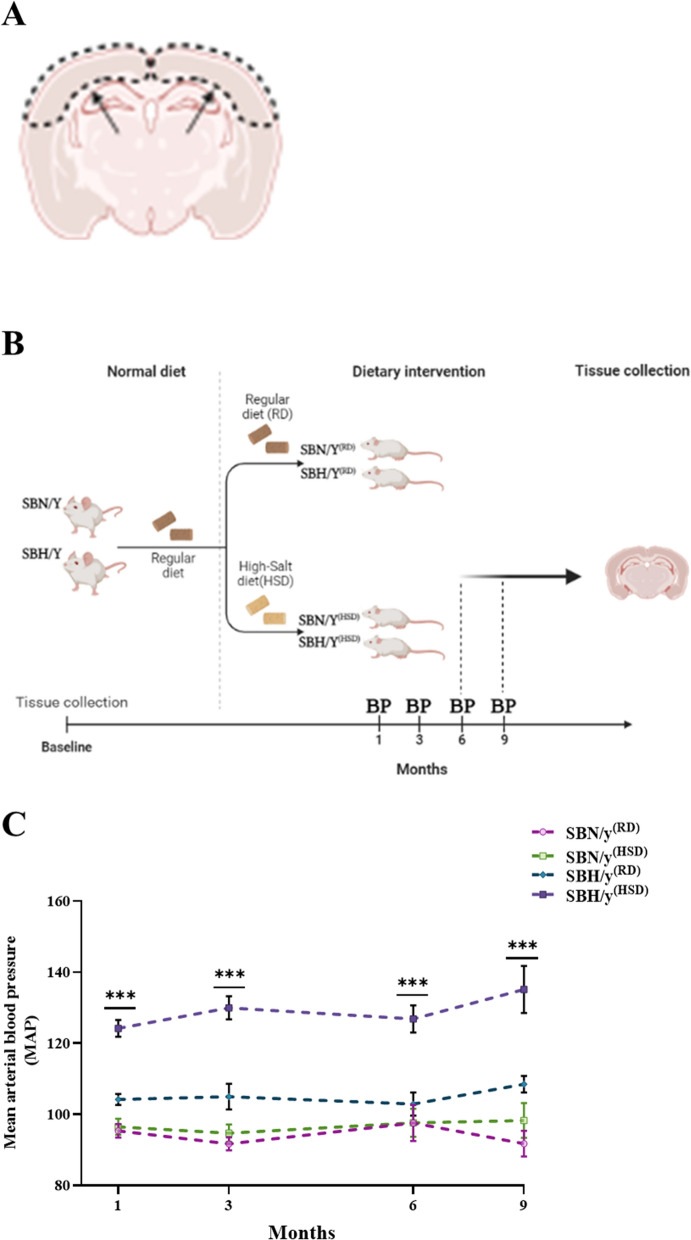
Schematic brain section and the experiment timeline. **A** Schematic coronal brain section of the parietal cortex region where immunohistochemistry staining was done; the black arrows indicate the area where the micrographic picture was taken and analyzed. **B** the timeline illustrates the experimental procedures across 9 months of the study; the Animals were separated into four groups: SBN/y^(RD)^ and SBH/y^(RD)^, provided a regular rat diet (RD), SBN/y^(HSD)^, and SBH/y^(HSD)^ provided a high salt diet (HSD). Blood pressure was measured at 4 points: 1, 3, 6, and 9 months after initiation of RD or HSD. Animals were sacrificed at three time points: baseline, 6 months, and 9 months on RD or HSD. **B** MAP of the four groups (N = 4–17, per group). Mean ± SEM. One-way ANOVA was followed by Tukey’s multiple comparison test. ^***^P < 0.001, SBH/y + vs other groups

Three-month-old males were salt-loaded by feeding standard rat chow enriched with 8% NaCl (Cat. TD.92012, Harlan, Israel). SBH/y fed a high salt diet invariably develops hypertension, whereas SBN/y fed the same diet does not exhibit an elevation in blood pressure [31, 32]. Therefore, in order to examine the effect of salt-sensitive hypertension on molecular changes in the brain, four groups of animals were studied: SBH/y^(HSD)^ provided a high salt diet (HSD) as the experimental hypertensive group, SBH/y^(RD)^ provided a regular rat diet (RD) as the hypertension-prone salt-control group, SBN/y^(HSD)^ provided HSD as the hypertension-resistant salt-control group and SBN/y^(RD)^ on regular diet as the strain control group (Fig. 1B). In addition, rats were provided RD prior to switching to HSD.

### Blood pressure measurements

Blood pressure was measured using a non-invasive tail-cuff method (CODA^™^, Kent Scientific Corporation, Torrington, CT, USA). Briefly, rats were placed in restraint tubes and left for 20 min to warm (tail skin surface temperature ~ 30 °C) on the instrument warming platform, and a pressure transducer was placed on the rat's tail. In order to prevent stress effects, rats were allowed to habituate to this procedure for 5 days before mesurments were performed. Final measurements were averaged from 10 consecutive readings obtained from each rat. The Coda^™^ instrument displays diastolic, systolic, and mean arterial blood pressure (MAP). However, we used the mean arterial blood pressure (MAP) for the analyses.

### Staining of tissue sections

Animals from each group were sacrificed at three time points: At baseline, after 6 and 9 months of feeding with HSD or RD. Rats were deeply anesthetized, sacrificed, and transcardially perfused with heparinized saline, 10% sucrose in buffered saline, and 4% buffered formaldehyde for 48 h. Blocks containing the cortex were embedded in paraffin. Consecutive 5 μm sections were cut using a microtome and placed onto IHC adhesive glass slide (TM-1190 TOMO, MATSUNAMI). Slides were then deparaffinized and subjected to heat-induced epitope retrieval using OmniPrep (#ZUC067-100, ZYTOMED SYSTEMS) according to manufacturer instructions. In order to minimize background immunofluorescence (IF), Background Buster (#NB306-50, INNOVEX) was added onto the slide for 30 min at room temperature, followed by three cycles of wash buffer for two minutes each, then incubated overnight at 4 ºC in a humidity chamber with one of the first antibodies: anti-Atg4a (1:200; #EPR4122, Millipore); anti-MAP1LC3A/B (1:200; #SAB4300, Novus Biologicals); cleaved anti-Caspase-3 (1:100; #CPP324, Zytomed System); Anti HDAC2 (1:250; #Y461, Millipore); anti-HAT1 (1:250; #11432, Proteintech Group); anti-eIF2α ~ P (1:200; TA343946 OriGene Inc.) Slides were rinsed 3 times using wash buffer and incubated for 1 h with a secondary antibody anti-rabbit/mice 594 (1:150; Bethyl Laboratories, Inc.), followed by three cycles of wash buffer for two minutes each. Next, the sections were mounted with Fluoromount-G^™^ with DAPI (eBiosciences), after which the slide was dried, and a glass cover was affixed and sealed with glue.

### Immunofluorescent staining and microscopy

Microscopic analyses were performed using the Eclipse Ci microscope (Nikon Corp., Japan). In addition, Cortical images (Fig. 1A) of 10 fields were captured by the Nikon DS-Ri1 camera (Nikon Corp., Japan) with the same microscope settings and exposure time and analyzed using the Image J software 1.46 by an observer blinded to the condition.

### Gene expression analysis

RNA from cortical sections (Fig. 1A) was extracted by the miRNeasy Mini Kit (QIAGEN, USA) according to kit instructions. RNA was converted to complementary DNA (cDNA) by qScript^™^ cDNA Synthesis Kit (Quanta Bioscience^™^). Cortical genes were assayed in triplicates for each time-point for each animal using the Real-Time PCR cycle thermal protocol by StepOnePlus real-time PCR instrument (Applied Biosystems) and the Quanta BIOSCIENCE PerfecTa^®^SYBR^®^ Green FastMix^®^, ROX^™^ kit. PCR cycle thermal protocol was: 95 ºC for 3 min, 95 ºC for 15 s, 60 ºC for 1 min. The relative expression of Atg4a, LC3A, Caspase-3, eIF2α, ATF4, and mTORC1 normalized to glyceraldehyde-3-phosphate dehydrogenase (GAPDH) and was calculated using the ΔCt method. The Gene primer sequences are listed in Additional file 1: Table S1.

### Data analysis

Statistical analyses were performed using IBM SPSS version 26 and GraphPad Prism. All data were expressed as Mean ± SEM. Differences between groups were assessed by Student *t*-test, one-way ANOVA, and three-way ANOVA. Differences were determined Post hoc by Tukey’s test and Student *t*-test when significant main effects or interactions were detected. The significance level was set at *p* < 0.05.

## Results

### Blood pressure

As of 1 month after initiation of HSD, MAP of SBH/y^(HSD)^ was significantly higher at each time point compared to SBN/y^(RD/HSD)^ and SBH/y^(RD)^ (F_(3,53)_ = 39.68, *P* < 0.001; F_(3,40)_ = 43.73, *P* < 0.001; F_(3,22)_ = 12.57, *P* < 0.001; F_(3,16)_ = 13.30, *P* < 0.001). There was no significant difference in MAP between SBN/y^(HSD)^ group and SBN/y^(RD)^ and SBH/y^(RD)^ treated with RD (Fig. 1C; See Additional file 1).

### Regulation of gene transcription

**Fig. 2:**
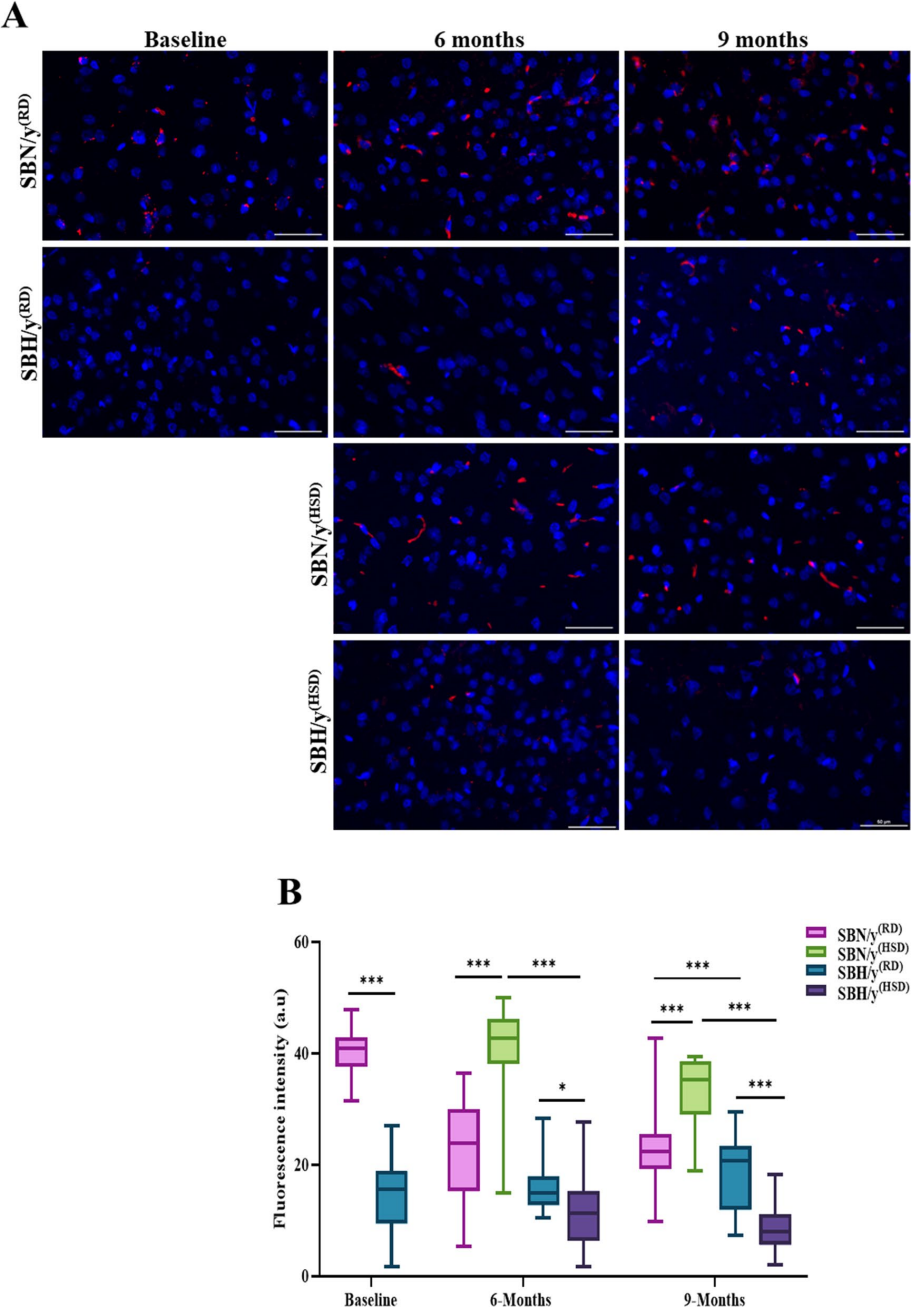
Brain cortical levels of Histone deacetylase 2 (HDAC2). Representative merge images of DAPI–DNA blue staining of cells' nuclei and HDAC2 protein-red staining in SBN/y and SBH/y s at three time points: baseline, 6 months, and 9 months on RD or HSD. *Scale bar: 50 µm*. **B** IF analysis of cortical HDAC2 protein ratio level (N = 6–7, per group). Mean ± SEM. Three-way ANOVA was followed by Tukey’s multiple comparison test. ^*^P < 0.05, ^***^P < 0.001

At baseline and before the dietary intervention, the expression HDAC2 in SBH/y was significantly lower than in SBN/y (*P* < 0.001). Subsequently, strain and diet type were significantly affected, but not diet duration, on the expression of HDAC2 (Fig. 2A, B). At each time point, the expression of HDAC2 in SBH/y^(HSD/RD)^ groups was significantly lower than in SBN/y^(HSD/RD)^ groups (Fig. 2A, B). Notable, HSD, compared to RD, increased the expression of HDAC2 in the SBN/y strain and decreased the expression in the SBH/y strain (Fig. 2A, B).

**Fig. 3:**
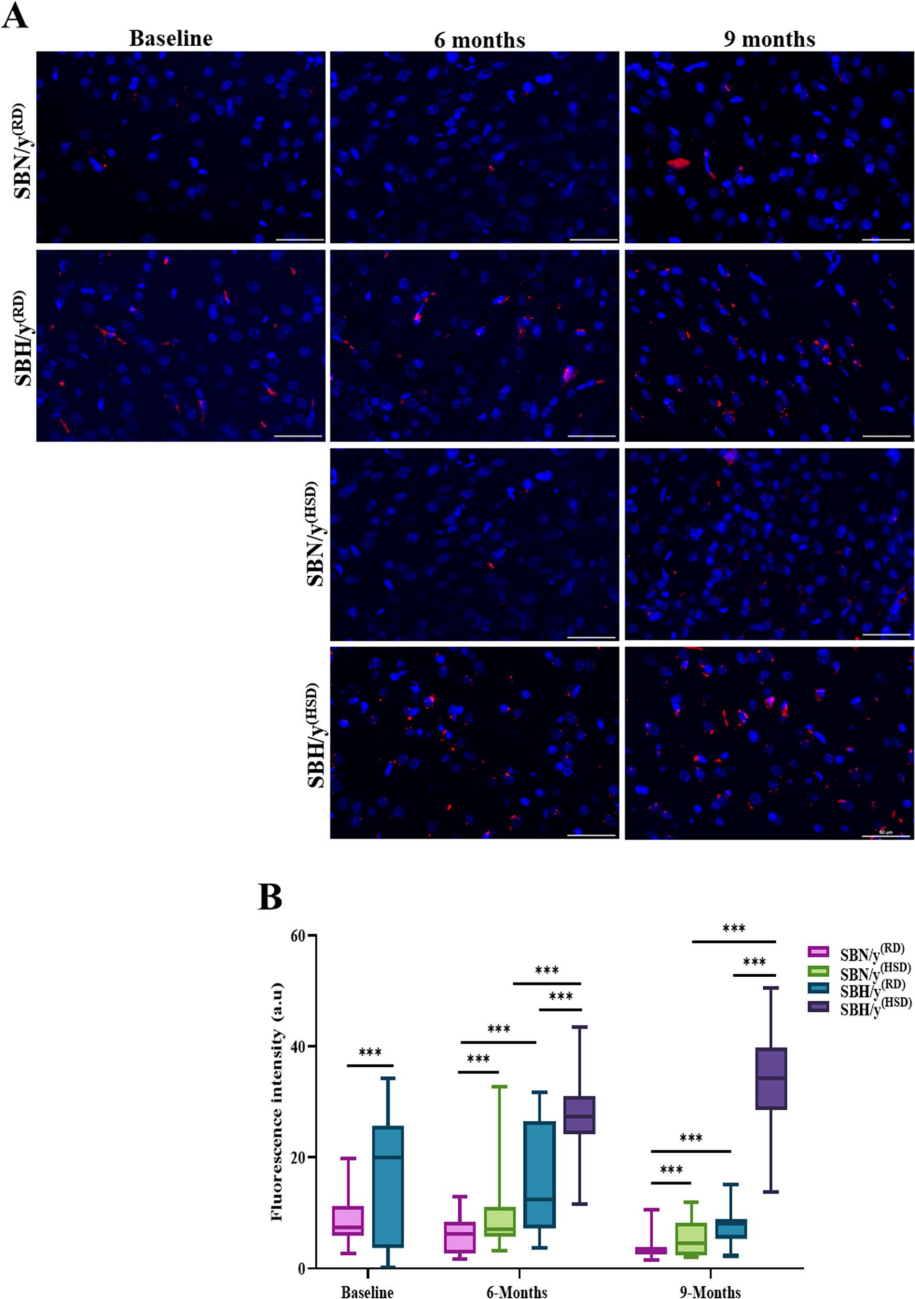
Brain cortical levels of Histone acetyl transferase-1 (HAT1). **A** Representative merge images of DAPI—DNA blue staining of cells' nuclei and HAT1 protein-red staining in SBN/y and SBH/y at three-time points: baseline, 6 months, and 9 months on RD or HSD. *Scale bar: 50 µm*. **B** IF analysis of cortical HAT1 protein ratio level (N = 6–7, per group). Mean ± SEM. Three-way ANOVA was followed by Tukey’s multiple comparison test. ^***^P < 0.001

The balance between acetylation by acetyltransferases and deacetylation by histone deacetylases regulates the gene expression levels, and this balance is necessary for cellular homeostasis [33–35]. Therefore, we measured the expression of Histone Acetyltransferase 1 (HAT1) to determine the effect of HSD on the acetylation process among rats with a genetic predisposition to hypertension. At baseline and prior to dietary intervention, the expression of HAT1 in SBH/y was significantly higher than in SBN/y (*P* < 0.001). Subsequently, there was a significant effect and interaction of strain and diet type, but not diet duration, on the expression of HAT1 (Fig. 3A, B). At each time point, the expression of HAT1 in SBH/y^(HSD/RD)^ groups was significantly higher than in SBN/y^(HSD/RD)^ groups (Fig. 3A, B). Notable, HSD, compared to RD, increased the expression of HAT1 in both strains (Fig. 3A, B).

### Autophagy in the brain cortex

**Fig. 4:**
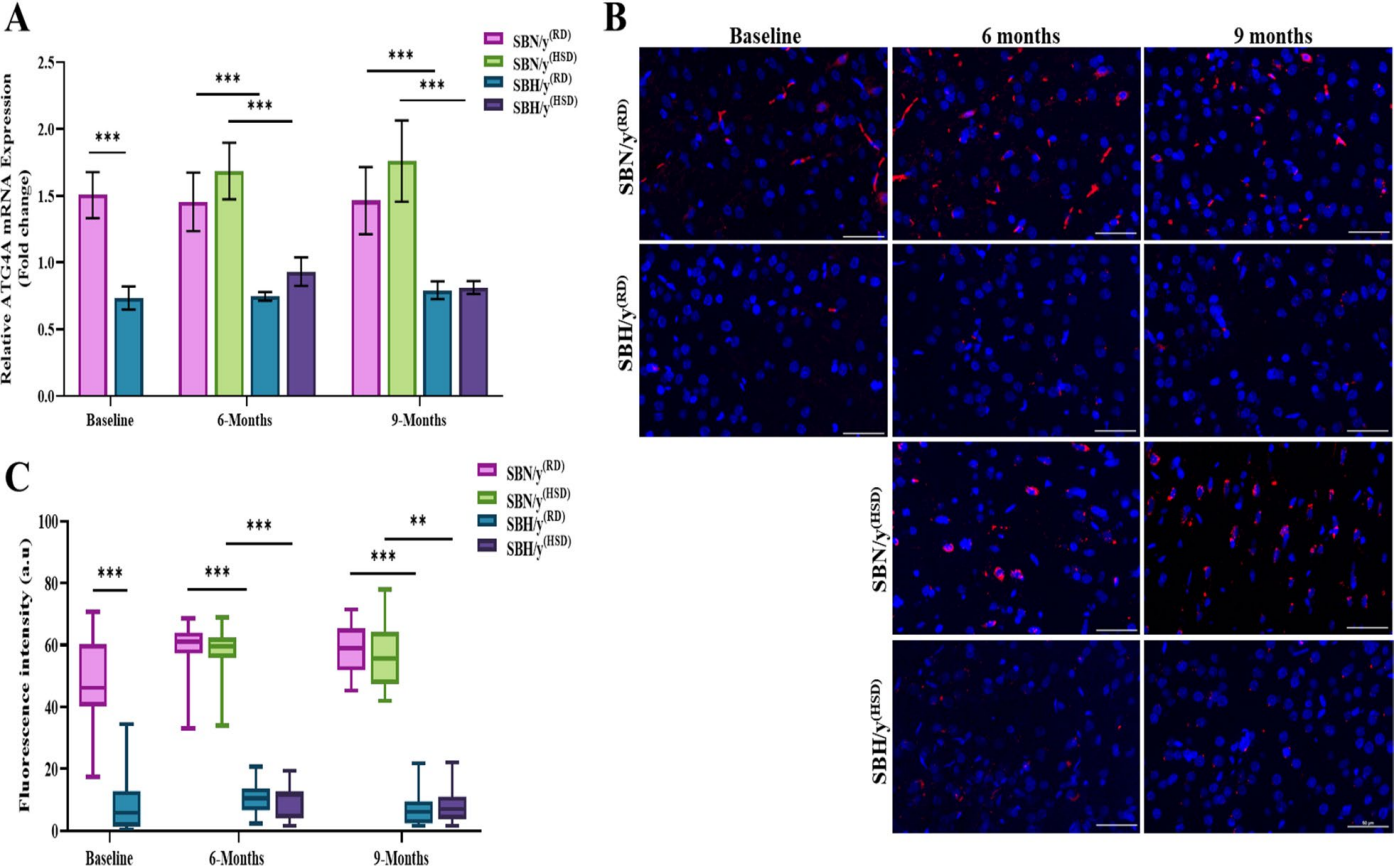
Cortical Cysteine protease (ATG4A) mRNA and protein level. **A** Cortical Cysteine protease (ATG4A) mRNA (N = 6–8 per group). **B** Representative merged images of DAPI—DNA blue staining of cells' nuclei and Atg4a protein-red staining in SBN/y and SBH/y at three-time points: Baseline, 6 months, and 9 months on RD or HSD. *Scale bar: 50 µm*. **C** IF analysis of the cortical ATG4A protein level (N = 6–7 per group). Mean ± SEM. Three-way ANOVA was followed by Tukey’s multiple comparison test. ^**^P < 0.005; ^***^P < 0.001

At baseline and prior to dietary intervention, the expression of ATG4A at the mRNA and protein levels was lower in SBH/y than in SBN/y (*P* < 0.001; Fig. 4A–C). The diet type (HSD or RD) or duration had no significant effect at 6 and 9 months on the expression of ATG4A at the mRNA and protein level in either strain (Fig. 4A–C). Nonetheless, at each time point, SBH/y^(HSD/RD)^ expressed lower mRNA and protein levels of ATG4A compared to the SBN/y^(HSD/RD)^ group (*P* < 0.001; Fig. 4A–C).

**Fig. 5:**
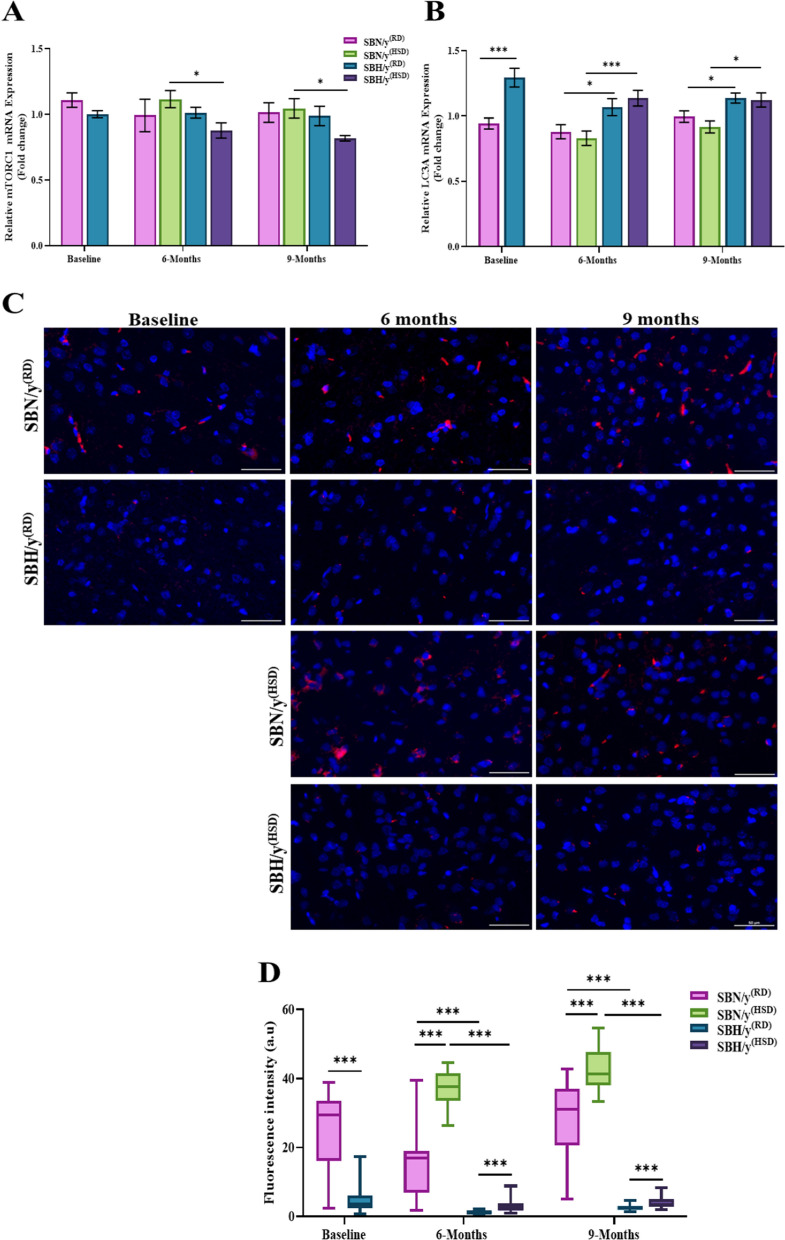
Brain cortical mTORC1 and LC3A expression at mRNA and protein level. **A** Cortical mTORC1 mRNA expression (N = 5–8 per group). **B** Brain cortical LC3A mRNA expression (N = 5–8 per group). **C** Representative merged images of DAPI—DNA blue staining of cells' nuclei and LC3A/B protein -red staining in SBN/y and SBH/y groups at three-time points: Baseline, 6 months, and 9 months on RD or HSD. *Scale bar: 50 µm*. **C**, **D** IF analysis of the cortical LC3A/B protein expression (N = 6–7 per group). Mean ± SEM. Three-way ANOVA was followed by Tukey’s multiple comparison test. ^*^P < 0.05; ^***^P < 0.001

Atg4A converts microtubule-associated protein 1 light chain 3 (MAP1LC3)-LC3 in the autophagy process to the cleaved form-LC3A/B. Here, we measured changes in the expression of LC3A at the mRNA level and the post-translation modification level (Cleaved form-LC3A/B). Prior to salt loading, high expression of LC3A at the mRNA level in SBH/y compared to the SBN/y strain (*P* < 0.001; Fig. 5B) was found. The diet type (HSD or RD) or duration, however, had no significant effect at 6 and 9 months on the expression of LC3A at the mRNA level (Fig. 5B). Nonetheless, at each time point, SBH/y^(HSD/RD)^ groups expressed higher levels of LC3A mRNA compared to the SBN/y^(HSD/RD)^ groups (*P* < 0.001; Fig. 5B). We also measured changes in LC3A/B at the post-translation modification level (Cleaved form). Before salt diet loading, we found lower expression of LC3A/B among SBH/y compared to the SBN/y strain (*P* < 0.001; Fig. 5C, D). In both strains, there was a significant effect of diet type and duration (6 months, 9 months) on the expression of LC3A/B (*P* < 0.001; Fig. 5C, D). After 6 and 9 months, SBH/y^(HSD)^and SBN/y^(HSD)^groups expressed high levels of LC3A/B protein compared to SBH/y^(RD)^ and SBN/y^(RD)^ groups (*P* < 0.001; Fig. 5C, D). We also found that after 9 months, all groups (SBH/y^(RD)^, SBH/y^(HSD)^, SBN/y^(RD)^, and SBN/y^(HSD)^) expressed more LC3A/B protein than after 6 months (*P* < 0.001; Fig. 5C, D). Nevertheless, SBH/y provided HSD or RD expressed lower levels of LC3A/B protein at each time point than SBN/y provided a similar diet (*P* < 0.001; Fig. 5C, D).

To further explore autophagy in our model of salt-sensitive hypertension, we examined the expression of the negative regulator of autophagy—mTORC1. Prior to salt-loading, we found no significant difference in mTORC1 mRNA expression between SBH/y and SBN/y (Fig. 5A). However, after 6 and 9 months of the dietary intervention, however, we detected a significant effect on salt loading in SBH/y. Expression of mTORC1 was lower in SBH/y^(HSD)^ than in SBN/y^(HSD)^ (*P* < 0.05; Fig. 5A), but there was no difference in the expression between SBH/y^(RD)^ and SBN/y^(RD)^.

### The effect of genetic predisposition to salt-sensitive hypertension on the stress response in the brain cortex

**Fig. 6:**
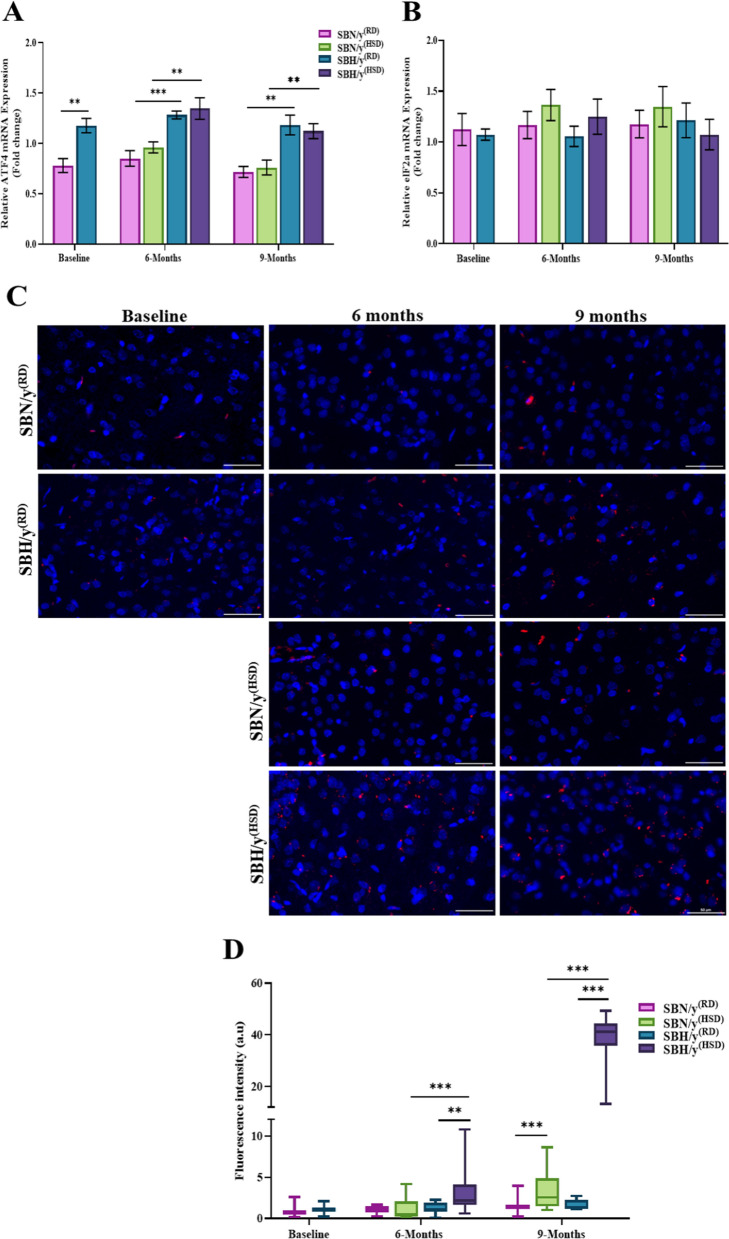
Cortical ATF4 and eukaryotic translation initiation factor 2 Alpha (eIF2α) at the mRNA level and phosphorylated (eIF2α ~ P) at the protein level. **A** Brain cortical ATF4-mRNA expression (N = 6–7 per group). **B** Brain cortical eIF2α-mRNA expression (N = 5–6 per group).** C** Representative merged images of DAPI—DNA blue staining of cells' nuclei and eIF2α ~ P protein-red staining in SBN/y and SBH/y groups at three-time points: Baseline, 6 months, and 9 months after RD or HSD. *Scale bar: 50 µm*. **D** IF analysis of the cortical eIF2α ~ P protein expression (N = 6–7 per group). Mean ± SEM. Three-way ANOVA was followed by Tukey’s multiple comparison test. ^**^P < 0.005; ^***^P < 0.001

Prior to salt loading, we found a higher expression of ATF4 at the mRNA level in SBH/y compared to SBN/y (*P* < 0.01; Fig. 6A) but no difference in the expression of eIF2a mRNA between SBH/y and SBN/y (Fig. 6B). We then examined the effect of an HSD on ATF4 and eIF2a mRNA expression. We found no effect of strain, diet type, and diet duration on eIF2a mRNA expression (Fig. 6B) but found a higher expression of ATF4 in SBH/y^(HSD/RD)^ compared to SBN/y^(HSD/RD)^ at each time point (*P* < 0.01; Fig. 5A). Diet type or duration did not affect ATF4 mRNA expression in both strains. Thus, animals of both strains on HSD and RD expressed similar levels of ATF4 mRNA (Fig. 6A).

Since salt-loading affects protein activation by post-translational modifications, we examined whether cortical eIF2α phosphorylation is altered during HSD. Before salt diet loading, there was no significant difference in the level of eIF2α phosphorylation between SBH/y and SBN/y (Fig. 6C, D). Subsequently, there was a significant interaction of strain, diet type, and diet duration on the level of eIF2α phosphorylation. At each time point, SBH/y^(HSD)^ expressed higher levels of eIF2α phosphorylation than SBH/y^(RD)^ group or SBN/y^(HSD/RD)^ groups (*P* < 0.05; Fig. 6C, D). After 9 months of HSD, SBN/y^(HSD)^ also expressed higher levels of eIF2α phosphorylation compared to SBN/y^(RD)^ and SBH/y^(RD)^ groups(*P* < 0.05; Fig. 6C, D). The effect of diet duration was noted in SBH/y^(HSD)^and SBN/y^(HSD)^ by elevated expression of eIF2α phosphorylation after 9 months compared to 6 months (*P* < 0.001; Fig. 6C, D).

### The effect of genetic predisposition to salt-sensitive hypertension on apoptosis and parimydial neuronal cell density in the brain cortex

**Fig. 7:**
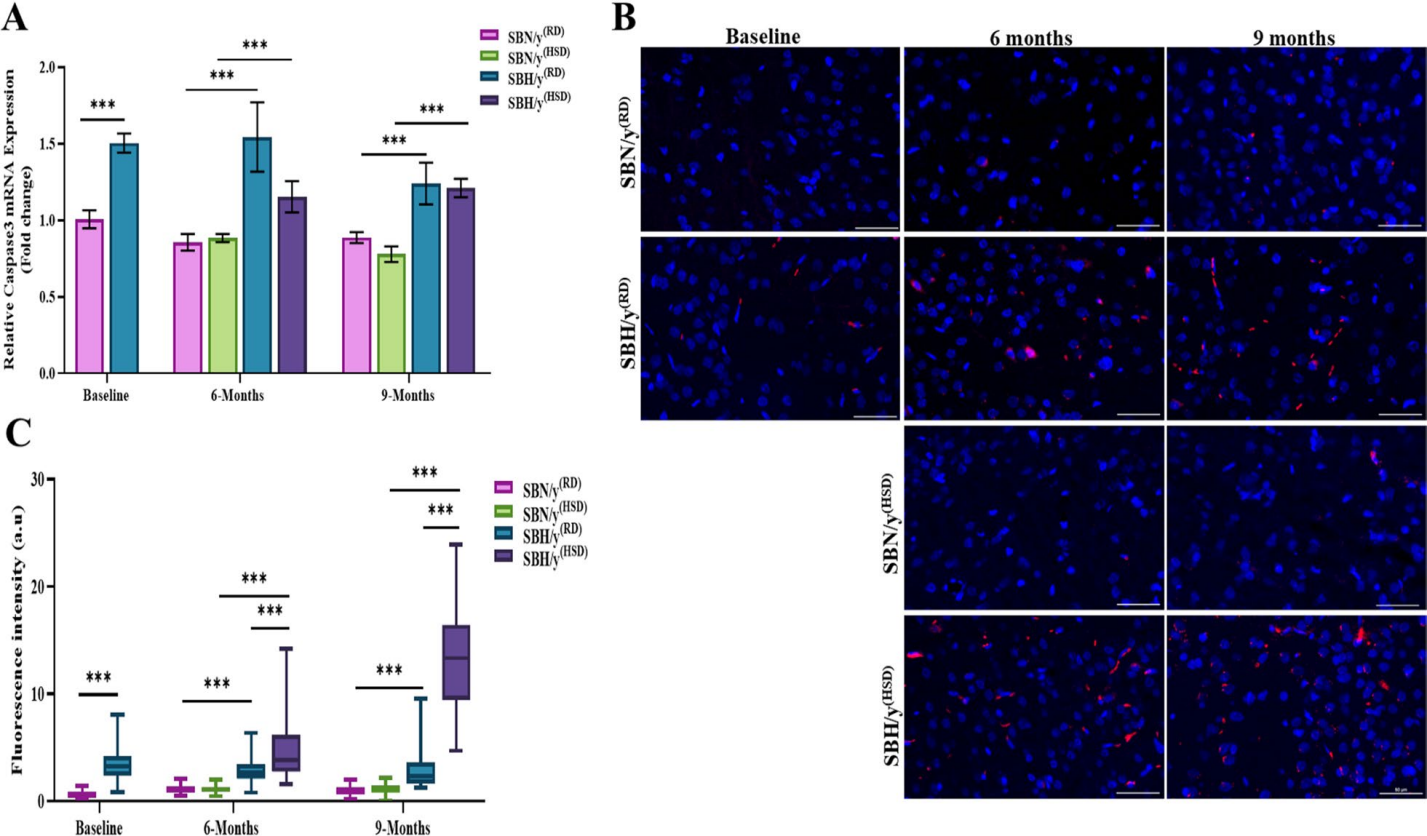
Cortical caspase-3 at mRNA level and activated caspase-3 (cleaved) at the protein level. **A** Brain cortical caspase-3 mRNA expression (N = 5–7 per group). **B** Representative merge images of DAPI—DNA blue staining of cells' nuclei and caspase-3 protein-red staining in SBN/y and SBH/y at three-time points: Baseline, 6 months, and 9 months on RD or HSD. *Scale bar: 50 µm*.** C** IF analysis of the cortical caspase-3 protein expression (N = 6–7 per group). Mean ± SEM. Three-way ANOVA was followed by Tukey’s multiple comparison test. ^***^P < 0.001

We found higher expression of caspase-3 at the transcription and protein level in SBH/y compared to the SBN/y strain (*P* < 0.001; Fig. 7A–C) prior to and during salt-loading. We next examined the effect of a salt diet combined with a genetic predisposition to salt-sensitive hypertension. At the transcription and protein levels, we found high expression of caspase-3 in SBH/y^(RD/HSD)^ groups compared to the SBN/y^(HSD/RD)^ groups at each time point (*P* < 0.001; Fig. 7A–C). In addition, there was a significant effect and interaction of strain, diet type, and diet duration at the protein level on the expression of caspase 3. At each time point, SBH/y^(RD/HSD)^ groups expressed higher levels of caspase-3 compared to the SBN/y^(RD/HSD)^ (*P* < 0.001; Fig. 7B, C). HSD increased the expression of caspase-3 protein only in SBH/y (*P* < 0.001; Fig. 7B, C). Finally, caspase-3 protein expression was elevated after 9 months compared to 6 months only in SBH/y^(HSD)^ (*P* < 0.001; Fig. 7B, C).

**Fig. 8:**
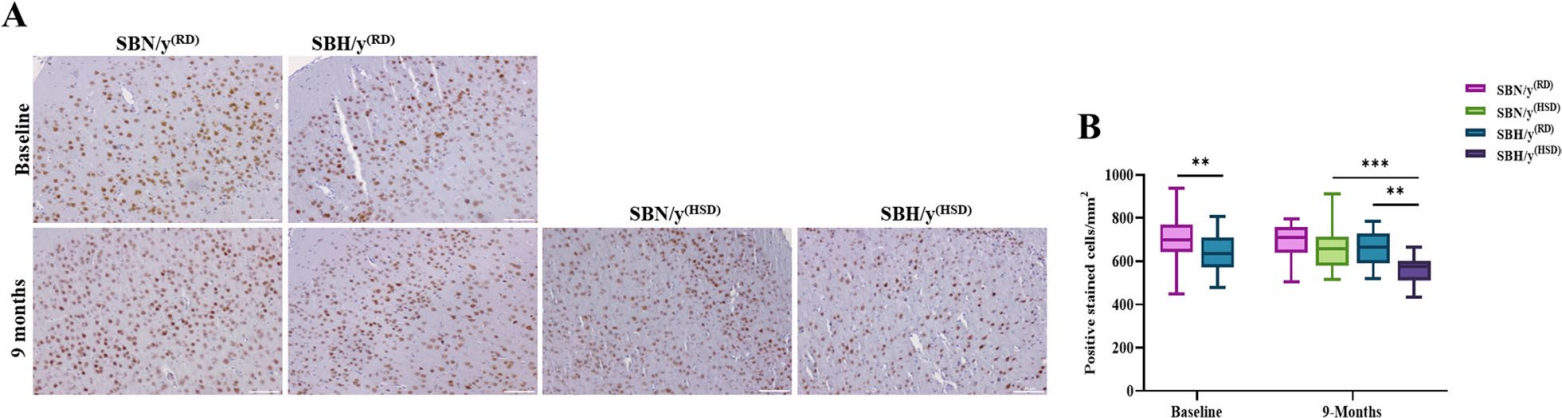
Cortical neuronal staining. **A** Representative images of brain cortical neuronal staining with anti-Neun in SBN/y and SBH/y at two-time points: Baseline, and 9 months on RD or HSD. *Scale bar: 100 µm*. **B** Number of positive neuronal cells/mm^2^ (N = 4–6 per group). Mean ± SEM. Two-way ANOVA was followed by Tukey’s multiple comparison test. ^**^P < 0.01,.^***^P < 0.001

Furthermore, we found lower density of pyramidal neuronal cell in SBH/y^(RD)^ compared to SBN/y^(RD)^ (*P* < 0.01; Fig. 8A, B), prior salt-loading. We next examined the effect of a salt diet combined with a genetic predisposition to salt-sensitive hypertension, we found lower density of pyramidal neuronal cell in SBH/y^(HSD)^ compared to SBN/y^(HSD/RD)^ and SBH/y^(RD)^ after 9 months of salt-loading (*P* < 0.01; Fig. 8A, B). However, there was no significant effect of time point on the neuronal density in SBH/y^(RD)^, and SBN/y^(RD)^ (*P* < 0.01; Fig. 8A, B).

## Discussion

**Fig. 9:**
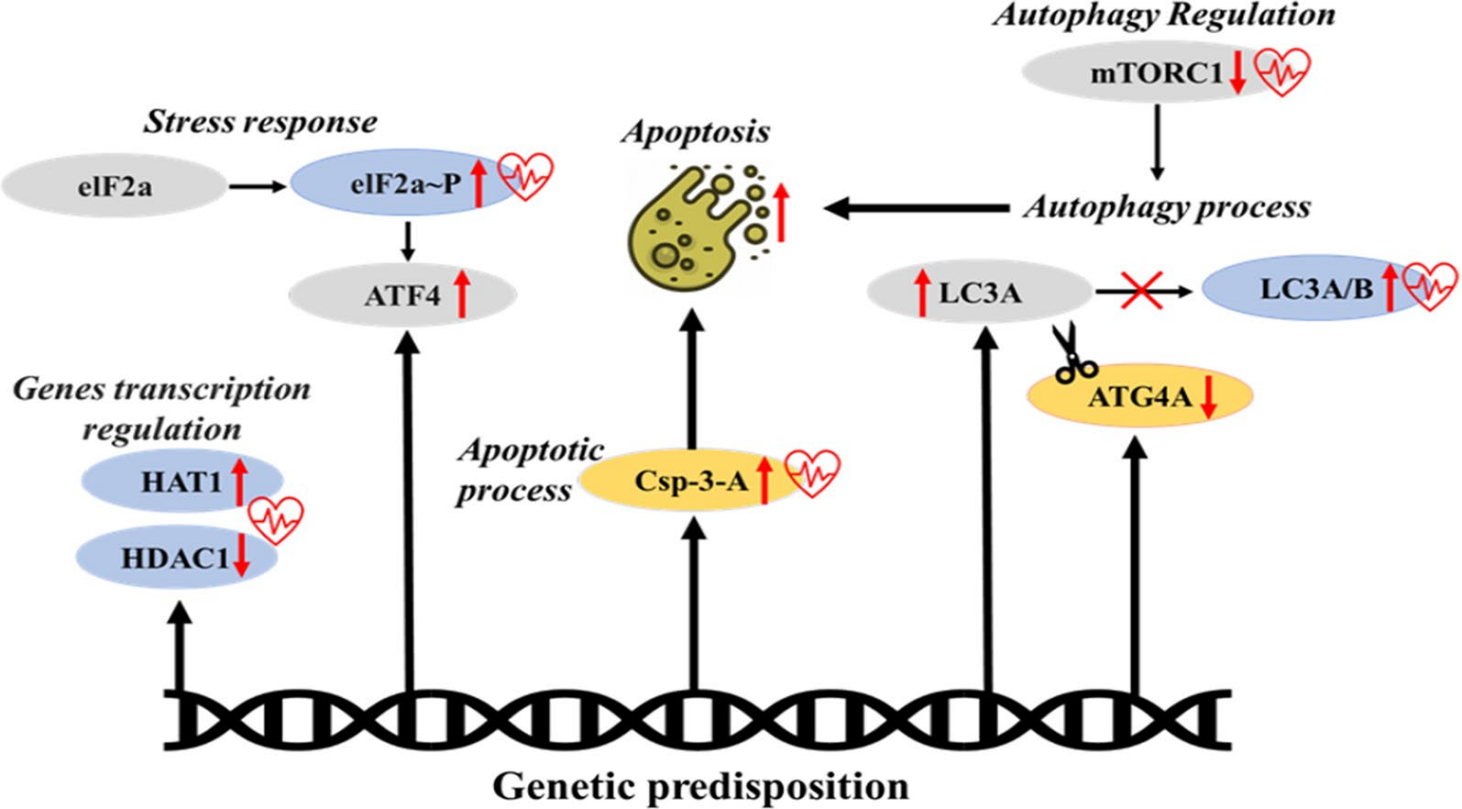
Model of the molecular neurodegeneration machinery in hypertension in the SBH/y^(HSD)^ strain after salt loading. Genetic predisposition to salt-sensitive hypertension is associated with cortical cellular dysfunction in gene transcription regulation, autophagy, stress, and apoptotic processes. These cellular dysfunctions are exacerbated by salt loading that induces concurrently hypertension. mRNA is denoted by gray, protein by light blue, and together by gold, hypertension effect denoted by a red heart

The current study highlights several cellular factors promoting cortical neurodegeneration and provides evidence for a neurodegenerative process secondary to hypertension in a genetic rat model (Fig. 9). The SBH/y strain has a genetic predisposition to develop high blood pressure when exposed to an HSD [32]. In SBH/y, salt sensitivity is genetically determined and expressed only after salt-loading, as spontaneous hypertension does not develop. Linkage analysis of the entire rat genome has previously identified three quantitative trait loci on rat chromosomes 1 and 17 that contribute significantly to salt-sensitive hypertension in SBH/y [32]. In the current study, consistent with previous reports, only SBH/y treated with a salt diet developed hypertension, whereas SBH/y provided RD and SBN/y irrespective of the diet remained normotensive.

A substantial body of evidence suggests that hypertension damages neuronal cells and promotes neurodegeneration [36–39]. The current results indicate that the genetic predisposition to hypertension is already associated with abnormality of cell processes such as regulation of gene transcription. Prior to salt loading, cortical HAT1 protein levels were higher in SBH/y than SBN/y, whereas HDAC2 was lower in SBH/y than SBN/y. Notably, the salt strengthened this pattern in the SBH/y strain. These results suggest that the enhanced histone acetylation may indicate transcriptional dysregulation in the brain cortex of SBH/y. Moreover, hypertension may mediate cell oxidative stress via mitochondrial dysfunction, which induces histone acetylation by a high level of HAT1, leading to apoptosis [40]. Nevertheless, the HSD diet has a negligible effect on the expression of HAT1 and HDAC2 in the cortex among the SBN/y strain, raising the possibility that HSD affects gene regulation even when there is no evidence of an increase in blood pressure.

Previous studies suggest that hypertension causes oxidative stress and inflammation in the central nervous system [41–43]. In response to the cellular stress, eIF2α phosphorylation represses global translation coincident with the preferential translation of ATF4 [29, 30]. In the current study, prior to salt loading, we found a high expression of ATF4 mRNA in SBH/y compared to the SBN/y group but without any other effect of salt loading. In addition, we found similar mRNA expression of eIF2α in both strains, SBH/y and SBN/y, with no significant effect of salt diet on the expression at the mRNA level. Given that hypertension affects protein activation by post-translational modifications, we examined whether cortical eIF2α phosphorylation is altered during the salt diet. The current result depicts an increase in the phosphorylation of eIF2α in rats with high blood pressure (SBH/y^(HSD)^).

It should be noted that after 9 months, there was a significant increase in the phosphorylation of eIF2α in the SBN/y group treated with a salt diet. However, the expression of eIF2α phosphorylated was higher in the SBH/y^(HSD)^ group than in the SBN/y^(HSD)^. These findings are supported by other stress studies where the induction of ischemia in neurons led to an increase in phosphorylation of eIF2α [44]. Our current results are consistent with the notion that chronic neuronal stress causes excessive phosphorylation of eukaryotic translation initiation factor 2α (eIF2α-P), which eventually impairs neuronal function and might contribute to the development of neurodegeneration [45]. Furthermore, the current result underscores that the combined effects of dietary salt intake and genetic predisposition to hypertension are more significant than the effects of each factor separately in provoking cellular stress.

Interestingly, the genetic predisposition to salt-induced hypertension was associated with a dysfunction in the autophagy process. Thus, lower levels of cortical Atg4a protease were found in SBH/y compared to SBN/y. However, salt loading has no further effect on the expression pattern. The autophagy process begins with converting microtubule-associated protein 1 light chain 3 (MAP1LC3)-LC3 to the cleaved form-LC3A/B by Atg4a [46]. At the mRNA level, we found that SBH/y expressed a higher level of LC3A than SBN/y, which was not affected by the salt diet. However, we observed that the decrease in the expression of Atg4a protease reflects in a low level of the cleaved protein form LC3A/B in SBH/y at each time point.

Additionally, in both strains, SBH/y and SBN/y, salt loading increased the level of the cleaved form LC3A/B, suggesting that salt stress affects the autophagy process. Consequently, the increase in expression of LC3A/B protein may indicate an interruption in cell function, requiring the activation of corrective mechanisms like autophagy to avoid accumulating cellular damage [40, 47]. In contrast, when the autophagy process is defective, as in SBH/y, salt loading could cause cellular damage that contributes to cell death. The regulation of the autophagy process was not directly associated with the genetic predisposition to hypertension. Before salt loading, we found a similar mRNA expression of the negative regulator of autophagy-mTORC1 in both strains, SBH/y and SBN/y. However, only in SBH/y did a significant change occur in the expression of mTORC1 after salt loading. Thus, we found a significant decrease in the expression of mTORC1 in SBH/y compared to SBN/y after salt loading. The reduction in mTORC1 expression in hypertension rats may suggest that autophagy regulation is disrupted [48].

The current results suggest that genetic predisposition to hypertension and hypertension are associated with dysfunction of the autophagy process. Following the current results, we speculate that the dysfunction of the gene regulation/expression under stress and the autophagy process might contribute to the accumulation of cellular damage, which in turn promotes apoptosis. To examine this hypothesis, we assessed the levels of cortical Caspase-3, which is known as the main caspase, activated during the apoptotic pathway. The current results support this hypothesis. Indeed, we found high expression of cortical caspase-3 at the mRNA and protein level (cleaved/ activated form), as well as a lower density of cortical neurons in the strain with a genetic predisposition to hypertension compared SBN/y strain. In addition, we observed only in SBH/y^(HSD)^ an increase in the expression of the activated form of cortical caspase-3, as well as a reduction in the density of cortical neurons following salt-loading and the development of hypertension. This result raises the possibility that the elevated level of cortical neuronal apoptosis/loss, partially mediated by hypertension, might promote neurodegeneration. These findings are compatible with previous reports showing that hypertension increases neuronal apoptosis [49].

Several limitations of our study should be acknowledged. First, given that there was no specific identification of the cell type in the cortex. Therefore, it is possible that the cellular dysfunctions that we described may have occurred in different types of cortex cells (e.g., neurons, microglia, astrocyte). However, in general, it is hard to ignore that impairment in each cortex cell type is related to dysfunction in the regular activity of the brain system that eventually might contribute to the development of neurodegeneration, indeed, we found a significant reduction in the density of cortical neurons in the strain with a genetic predisposition to hypertension especially following salt-loading. Thus, it’s possible that the cortical neuronal loss is a direct result of dysfunction of neuronal-cell, or a result of abnormal activity of other cortex cells such as astrocytes and microglia. Future studies could assess the combined effects of dietary salt intake and genetic predisposition to hypertension on specific types of brain cortex cells. Moreover, no functional or behavioral outcomes were measured for neurodegeneration in the current study. However, in hypertension, the brain suffers oxidative stress and inflammatory processes, which lead to neurodegeneration characterized by the progressive loss of neurons that most likely manifest in behavioral abnormality and impairment in learning and memory, which are some of the reported symptoms of neurodegenerative diseases [50, 51].

Despite these limitations, our results indicate that genetic susceptibility to hypertension is associated with cellular dysfunction that promotes a robust increase in apoptosis of brain cortex cells which probably contributes to the development of neurodegeneration. More critically, the cellular dysfunction is exacerbated by salt loading that concurrently induces the development of high blood pressure. The damaging effect of hypertension on neuronal cell function has been ascribed mainly to salt-loading [52, 53]. However, this notion is currently being challenged by our study's results suggesting that a high salt diet selectively affects cellular functions that exacerbate rather than initiate neurodegeneration in animals genetically predisposed to salt-sensitive hypertension. The genetic predisposition to cortical cellular dysfunction thus appears to fulfill a significant primary role in neurodegeneration, whereas hypertension probably plays a secondary role by accelerating the process, which is already prevalent in the genetically susceptible (Fig. 9).

## Supplementary Information

**Additional file 1: Table S1**. The Gene primer sequences. **Table S2.** Mean Arterial Blood Pressure.

## Data Availability

The datasets analyzed during the current study are available from the corresponding author upon request.
