# Supplementary material for: Predisposition to cortical neurodegenerative changes in brains of hypertension prone rats
Source: J Transl Med. 2023 Jan 27;21:51. doi: 10.1186/s12967-023-03916-y (PMC9881299; doi:10.1186/s12967-023-03916-y)
Supplement: Supplementary file 1 — Additional file 1: Table S1. The Gene primer sequences. Table S2. Mean Arterial Blood Pressure. [file 12967_2023_3916_MOESM1_ESM.docx]

**Table S1. The Gene primer sequences**

| **Primer name** | **F/R** | **Sequence 5' - 3'** | **Accession of mRNA: RefSeq** |
| --- | --- | --- | --- |
| Atg4a | F | ACAGTGCCTATAAAATAGGTGACGAG | NM_001126298 |
|  | R | AATCCCATAAAAGCTCCTTGACC |  |
| MAP1LC3A | F | AAGATCCCGGTGATCATCGA | NM_199500.2 |
|  | R | GCGCCGGATGATCTTGAC |  |
| mTORC1 | F | CCTCAGTGGTACAGGCACACA | NM_019906 |
|  | R | CTCAGACGCTCTCCCTCCAT |  |
| Caspase-3 | F | CTCTTCATCATTCAGGCCTGC | NM_012922.2 |
|  | R | AGTAACCGGGTGCGGTAGAGTA |  |
| ATF4 | F | CATGGCGCTCTTCACGAAA | NM_024403.2 |
|  | R | GCTGGTATCGAGGAATGTGCTT |  |
| eIF2α | F | GTTCCAAGCTGCATGAAGAGG | NM_001109339.1 |
|  | R | TTCTTGCCTCCTGTTTTGCAG |  |
| GAPDH | F | AGAGACAGCCGCATCTTCTTG | NM_017008.4 |
|  | R | GTAACCAGGCGTCCGATACG |  |

|  | SBN/y^(RD)^ | | SBN/y^(HSD)^ | | SBH/y^(RD)^ | | SBH/y^(HSD)^ | |
| --- | --- | --- | --- | --- | --- | --- | --- | --- |
| **Months** | Mean | SEM | Mean | SEM | Mean | SEM | Mean | SEM |
| 1 | 95.3 | 1.9 | 96.5 | 2.3 | 103.3 | 1.3 | 124.2 | 2.4 |
| 1.5 | 97.5 | 3.5 | 97.3 | 2.3 | 106.9 | 1.7 | 128.6 | 4.3 |
| 2 | 99.8 | 3.2 | 97.9 | 2.0 | 103.9 | 1.0 | 124.4 | 1.4 |
| 3 | 91.7 | 1.8 | 94.7 | 2.4 | 103.3 | 3.8 | 129.9 | 3.3 |
| 3.5 | 97.5 | 1.8 | 98.6 | 2.7 | 106.2 | 1.2 | 128.6 | 3.7 |
| 4 | 96.8 | 2.8 | 97.6 | 2.3 | 106.9 | 1.9 | 126.8 | 2.5 |
| 4.5 | 97.3 | 2.4 | 98.1 | 2.9 | 109.8 | 2.8 | 133.9 | 4.5 |
| 5 | 96.4 | 3.3 | 100.3 | 3.5 | 113.5 | 5.0 | 136.4 | 4.8 |
| 6 | 97.6 | 5.1 | 97.6 | 4.0 | 102.9 | 3.3 | 126.8 | 3.8 |
| 7 | 94.0 | 3.1 | 92.7 | 3.7 | 105.8 | 5.7 | 133.4 | 4.2 |
| 8 | 94.6 | 4.3 | 94.8 | 4.4 | 105.6 | 3.1 | 130.1 | 4.2 |
| 9 | 91.8 | 3.6 | 98.2 | 4.9 | 108.5 | 2.4 | 135.2 | 6.7 |

**Table S2. Mean Arterial Blood Pressure**
